# The effect of putrescine on space use and activity in sea lamprey (*Petromyzon marinus*)

**DOI:** 10.1038/s41598-022-22143-x

**Published:** 2022-10-17

**Authors:** Emily L. Mensch, Amila A. Dissanayake, Muraleedharan G. Nair, C. Michael Wagner

**Affiliations:** 1 Department of Fisheries and Wildlife, Michigan State University, East Lansing, MI 48824 USA; 2 Department of Horticulture, Michigan State University, East Lansing, MI 48824 USA

**Keywords:** Animal migration, Behavioural ecology, Conservation biology, Freshwater ecology, Invasive species

## Abstract

Fish use odor to avoid exposure to predation and disease. Harnessing these odors as repellents is proving useful for management initiatives that conserve native species or control invasive populations. Here, we evaluated the behavioral response of invasive sea lamprey to putrescine, a decay molecule that many prey organisms avoid. Putrescine is found in tissue extracts that contain sea lamprey alarm cue, and human saliva, two mixtures known to elicit flight and avoidance responses in migratory sea lamprey. We used two behavioral assays to evaluate metrics of repellency: behavioral preference (space use) and change in activity rates and found context-dependent results. In smaller assays with individual fish, we found that putrescine had no effect on sea lamprey activity but did induce avoidance. In larger assays with multiple animals, sea lamprey did not avoid putrescine. Our results also showed consistent changes in activity and avoidance behavior in sea lamprey exposed to alarm cue in the smaller assay, concluding that this design could prove useful as a high-throughput screening tool. We also investigated a novel odor identified in sea lamprey skin, petromyzonacil, and found no behavioral effects to this odor on its own or in synergy with putrescine. Our results show limited evidence that putrescine acts as robust repellent for sea lamprey and highlight the importance of environmental context when interpreting avoidance behavior in laboratory settings.

## Introduction

The sea lamprey (*Petromyzon marinus* L.) is a large ectoparasitic jawless fish whose suctorial mouth and rasping tongue inflicts significant damage to its host fish, often resulting in death. Valued in its native range in the northern Atlantic basin, an invasive population in the Laurentian Great Lakes threatens an estimated $7 billion fishery^1, 2^. Management of the invasive population currently relies on applications of two lamprey specific pesticides, or lampricides, to kill larvae in Great Lakes tributaries, combined with low-head dams that block access to suitable spawning habitats^2,3^. However, application costs are increasing, climate change models project an increase in sea lamprey growth and access to spawning habitats, concerns are rising that over-reliance on lampricides could lead to biological resistance, and there is societal pressure to remove dams and restore stream connectivity^4–7^. Additional control methods are needed to synergize with the lampricide program to maintain control efficacy and acceptability^7^.

Harnessing chemosensory cues may provide the key to unlocking innovative supplemental control methods^8^. For example, there is substantial interest in capturing invasive sea lamprey as they migrate into Great Lakes streams to spawn^9^. Upon entering rivers, the sea lamprey encounters a gauntlet of shoreline predators, and relies on chemical risk cues to survive passage to the spawning grounds^10^. In aquatic predator–prey dynamics, these predation-related odors can include predator kairomones, odors emitted directly from a predator, such as saliva, urine or feces^11,12^, disturbance cues volitionally released after the perception of risk often through pulses of urea^13,14^, and alarm cues inadvertently released from injured tissue after an attack, thereby alerting nearby receivers to the presence of an active predator^15,16^. Each of these predation related odors may alert nearby prey to the presence of predation risk, with alarm cues often eliciting the most powerful and consistent reactions as they provide more reliable species-specific evidence that a predator attack has occurred. Typical behavioral responses to alarm cues include area avoidance, increased use of shelter, and greater vigilance^17^. The sea lamprey shows significant avoidance behaviors when exposed to a conspecific alarm cue in laboratory settings^10,18–22^ and in natural streams^23–25^.

Field studies that deployed the sea lamprey alarm cue as a repellent to drive migrants towards trapping devices have proven promising. When alarm cue was introduced to one side of a stream channel, migrating sea lamprey were more likely to encounter a trap entrance located on the opposing side of the river whether traps were placed along dam faces or in the open river channel^24,25^. However, one barrier to using chemosensory cues in a repellent formulation is habituation, which occurs when an organism’s behavioral response to a stimulus diminishes after frequent or continuous exposure^26,27^. Sea lampreys are known to habituate to their alarm cue when continuously immersed for 4 h^28,29^. Applying a rotating “menu” of repellent formulations could provide a means to prevent habituation to alarm cue in management settings. Alternatively, a risk cue that is not part of the alarm cue could be used to induce dishabituation to the alarm cue, causing spontaneous recovery of the avoidance behavior^29^.

One class of potential repellents that could augment alarm cue includes decay odors. Among these odorants is putrescine, is a small aliphatic diamine produced by animal tissue decay. It generates an odor that triggers strong yet varying behavioral responses in animals^30^. This distinctive “death scent” is repulsive and elicits starkly adverse responses in some species, especially prey species, likely as an adaptive response to avoid predation risk or disease contagion^31^. Humans exposed to putrescine increase vigilance, area avoidance, and hostility as threat management behaviors^32^. Zebrafish (*Danio rerio*) exhibit avoidance and defensive behaviors when exposed to putrescine^30,33^, and cortisol levels significantly increase, indicating stress^33^. Putrescine induces the burial of conspecific hooded rats (*Rattus norvegicus domestica*)^34^ likely to prevent risks associated with corpses such as scavenger attraction or pathogen exposure^35,36^. Conversely, putrescine can be attractive for certain predators or scavengers, where an odor of death may indicate an available meal. Food scented with putrescine and a similar decay odor, cadaverine, are preferred by hooded rats^37^. These compounds also increased feeding behavior three-fold in goldfish (*Carassius auratus*)^38^. Consequently, responses to putrescine appear to depend on the ecological role of the receiver: prey seek to avoid areas scented with putrescine to reduce the likelihood of encountering scavenging predators that are attracted by the very same odorant.

Putrescine has been identified in extracts of sea lamprey tissues that contain the animal’s alarm cue^39^. It is also the most abundant amine in human saliva^40^. With sudden exposure, human saliva can elicit a powerful flight response in sea lamprey^41^. In laboratory raceway studies, human saliva induces avoidance in sea lamprey^42^, and combining saliva with alarm cue increased the potency of the response^10,43^. One explanation for this finding is the two cues act in an additive manner, providing the organism with an improved risk assessment through sensory complementation^44^. Here, the alarm cue may provide information that a conspecific injury has occurred, while saliva provides information of the proximity of a mammalian predator^10^. Conversely, if putrescine is a component of both human saliva and the alarm cue, the observed increase in avoidance may be due to threat-sensitivity, where an increased concentration of the cue provides information of a larger level of risk and thus leads to a more intense response^45^.

The sea lamprey is a solitary nocturnal migrant that chooses streams emitting the odor of conspecific larvae (i.e., larval cue) that advertises the presence of suitable spawning and rearing habitats^46–49^. As the animal transitions from the open, deep environment of nearshore waters into the relatively narrow, shallow bounds of rivers, it is exposed to a new suite of nocturnal shoreline predators (mammals, reptiles) that may be difficult to detect^50,51^. Shifting from parasite to prey, the sea lamprey ceases feeding prior to its upstream spawning migration and relies on stored energy to complete the journey. This energy should be conserved when making navigational choices. In rivers, this could involve moving along the shallow margins where water velocities are typically lower. However, because the sea lamprey reproduces only at the end of its life, avoiding predation should also be a high priority, and shorelines are risky zones. These choices should therefore be dependent on perception of the imminence of threat and adjusted to the likelihood of an attack. For migrating sea lamprey, this ‘predatory imminence continuum’ may be organized around spatial and temporal variations in odors that reveal varying predation risk^52,53^. Thus, we hypothesized that putrescine is behaviorally relevant to sea lamprey in one or more of three contexts that occupy different regions of the predatory imminence continuum. We predicted alarm cue would elicit the highest anti-predator behavioral response because it gives reliable information about both the presence of an active predator and an attack on a conspecific (or closely related) animal. If putrescine was a component of a salivary kairomone, it may elicit an intermediate response as it provides evidence of a predator, but no evidence of its activity or of a recent attack. Finally, if putrescine is perceived as a decay odor, we expected a weaker response as it provides evidence of death, but due to the ubiquity of “rot” compounds in nature, there is no information about the nature of the death or immediate risk through predator presence. However, as decay odors can attract mammalian predators, especially scavengers^37,38^, sea lamprey may avoid it to reduce the likelihood of encounters that could result in attack.

In the present study, we examined whether putrescine may prove useful as a supplementary repellent for use in sea lamprey management through a sequence of two experiments over two years. First, we examined the responses of individual sea lamprey to putrescine in a small behavioral arena, observing evidence of avoidance. To further explore the role of putrescine in sea lamprey anti-predator behavior, we next tested if groups responded similarly to putrescine in a larger laboratory raceway across three treatments: (1) putrescine at observed concentrations in sea lamprey skin, (2) putrescine in combination with a novel molecule identified in sea lamprey alarm cue extracts that may label the putrescine as coming from conspecifics, and (3) putrescine at an increased concentration. In each case, we compared the magnitude of responses to that observed from exposure to alarm cue and a neutral control.

## Methods and materials

### Study design

To understand the role of putrescine on repellent behavior, we tested the activity of sea lamprey exposed to putrescine treatments in two behavioral assays. Experiment 1 examined single sea lamprey behavioral responses to a solvent control (N = 18), alarm cue derived from full body (N = 13) or skin tissues (N = 19), putrescine (N = 10), and a novel compound observed in sea lamprey skin, petromyzonacil^39^ (N = 19) in a high throughput individual assay in June 2020. In the following year, we further examined the role of putrescine in Experiment 2 by exploring the effect of three treatments on sea lamprey behavior in a larger multi-animal assay (each trial consisting of 10 fish) of the type described in Bals & Wagner^18^ between June and July 2021. First, we tested putrescine (N = 10) at the observed concentration within the skin extract. Next, we tested the same concentration of putrescine in combination with petromyzonacil (N = 10). Finally, we tested a high molarity treatment (10^–1^ M; N = 5) to determine whether response was related to odor concentration. The responses to these formulations were compared to the alarm cue extracted from carcasses (N = 10) or skin (N = 15), and a solvent control (N = 10).

### Odor collection and preparation

Per methods laid out in Wagner et al.^20^, full body alarm cue was collected from whole carcasses of male and female sea lamprey that naturally senesced during captivity. Senesced animals were immediately collected from holding tanks and frozen at − 20 °C until used in odor extractions. Extracts derived from freshly deceased sea lamprey elicit comparable avoidance responses as those derived from live donors^18^. Odor was collected from nine sea lamprey total, weighing approximately 1450 g, in a Soxhlet apparatus (2.08 m, Ace Glass Inc., Vineland, New Jersey, USA) connected to a water-cooled Allihn condenser. Solvent reservoirs with 12 L capacity were loaded with a 50:50 mixture of 200 proof ethanol and deionized water and refluxed (75–80 °C) using a hemispherical mantle for approximately six hours, yielding 10.2 L of alarm cue extract. Extracts, cooled overnight, were then decanted followed by filtration through muslin, and stored at − 20 °C until experimental use.

Individual compounds (petromyzonacil and putrescine) were isolated from sea lamprey skin extracts per the methods of Dissanayake, Wagner and Nair^39,54,55^. Alarm cue derived from sea lamprey skin tissue was collected from male and female sea lamprey that naturally senesced during captivity. Skins were removed postmortem and kept at − 20 °C prior to extraction. Odor extracts were collected through Soxhlet extraction which was carried out using 80:20 EtOH:RO water and ethanol removed by rotary evaporation before lyophilization. Lyophilized extracts were stored at − 80 °C until use. Individual compounds were purified from the skin extract by chromatographic methods including preparative HPLC. Purified compounds were characterized by spectroscopic methods including NMR and MS analyses. For Experiment 1, putrescine and petromyzonacil were separately identified to be at 0.04% of the total skin extract, or 0.08 mg/L per extract of one skin. Putrescine and petromyzonacil odor solutions were prepared by dissolving 0.08 mg of each compound in 10 mL pure ethanol and followed by adding 990 mL solution of 50:50 DI H_2_O:EtOH to yield 1L solution in total. For Experiment 2, putrescine was prepared in the same way (resulting in a concentration of 10^–4^ M). The putrescine + petromyzonacil treatment was created by combining solutions of putrescine (0.08 mg in 10 mL) and petromyzonacil (0.08 mg in 10 mL) and bringing up the final volume to 1L with 980 mL of 50:50 DI H_2_O:EtOH mixture. The high concentration putrescine treatment (10^−1^ M) was made by dissolving 1.7 g dry putrescine in 10 mL ethanol and bringing it up to 1L with 990 mL 50:50 DI H_2_O:EtOH.

### Experimental subjects

All sea lamprey were collected through the U.S. Fish and Wildlife Service’s annual trapping operations in tributaries of Lake Huron (Cheboygan and Ocqueoc Rivers, Michigan, USA) or the channel connecting Lake Superior and Lake Huron (the St. Mary’s River). All fish were at the migratory sub-adult life stage and were transported to Hammond Bay Biological Station (HBBS) in tanks receiving continuous aeration. Experimental animals were sorted by sex, and only males were used because previous experiments demonstrated no difference in response to alarm cue between sexes in sexually immature migrants, and female sea lamprey decreased response during sexual maturation^18^. All fish were held in 1385 L round tanks that received continuous water flow sourced from Lake Huron (with a 100% exchange each 4 h) with supplemental aeration and a natural day-night light cycle. All animal care and procedures were approved by the Michigan State University Institutional Animal Care and Use Committee via permits AUF 02/16-015-00 and PROTO201900060. All methods were performed in accordance with the AFS/AIFRB/ASIH Guidelines for the Use of Fishes in Research^56^ and AVMA Guidelines for the Euthanasia of Animals^57^. The study is reported in accordance with ARRIVE guidelines^58^.

### Behavioral assay

**Figure 1:**
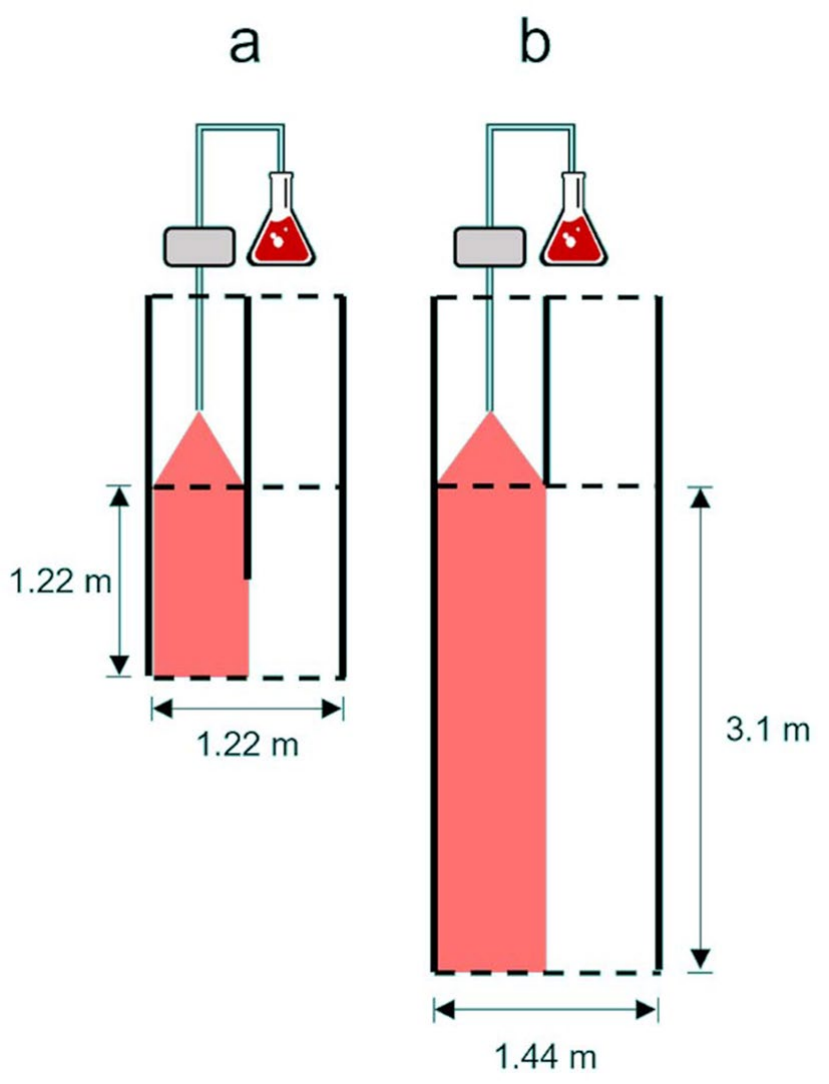
Schematic of experimental arenas: (**a**) small individual animal assay and (**b**) larger multi-animal assay, drawn to scale. Fish were introduced into the center of the area at the beginning of trials and were able to move freely throughout the arena over the course of the trial. Odor was introduced through one peristaltic pump before the stimulus observation period, and pump sides were switched after the completion of each trial. Dotted lines represent blocknets and thick black lines represent impervious and impassable partitions.

All experiments took place in two laboratory raceways at HBBS with dimensions 1.44 m × 12.2 m, and within either individual (Experiment 1; Fig. 1a) or in groups of 10 fish (Experiment 2; Fig. 1b) experimental arenas, described below. Trials were conducted in full darkness between 18:00 and 02:00 h during the spring spawning season, to approximate times and conditions of a typical nocturnal sea lamprey migration in streams. Water flowed into flumes from a head tank supplied directly from Lake Huron. Turbulence in each arena was reduced by placing baffles (rolled plastic mesh) at the upstream end. Two hours before experimental trials, subjects were visually inspected to ensure immature status and transferred to holding baskets constructed to allow water to constantly flow through and were held in round holding tanks until the beginning of the trial. All trials consisted of an acclimation period, a stimulus observation period where the odors were introduced. Odors were introduced into one-half of the experimental arena (left or right side), with the stimulus side alternating after each replicate. Peristaltic pumps (MasterFlex model 7533-20) released odor solutions, continuously stirred with a 2 cm magnetic stir bar to ensure a homogenous mixture, through PVC tubing at a fixed rate of 20 mL min^−1^. Dilutions were calculated based on raceway discharge measured by width, depth and velocity of the water, allowing for target solution dilution of 2:1,000,000 odor:lake water extract within the raceway. Separate sets of tubing were used for each odor treatment to ensure no cross contamination. Dye tests conducted prior to trials confirmed odor plumes were confined to the target half of the experimental arena. At the conclusion of each trial, each subject was removed from the arenas and total length (TL, cm) and wet weight (g) were recorded.

#### Experiment 1: Behavioral responses by individuals in a small-arena assay

Four individual test arenas were created by installing prefabricated 1.22 m × 2.44 m experimental two-choice arenas made from HDPE paneling into the raceways (two in each raceway as in Fig. 1a). The test area of each arena measured 1.22 m × 1.22 m, blocked off with mesh block nets. A 0.61 m panel of HDPE extended into the experimental arena to aid with stimulus partitioning. Video cameras (Lorex 8-Channel 4 K UHD NVR with 2 TB HDD) and infrared lights (4 5 MP Night Vision Bullet) were installed above each arena. Water temperature ranged from 11–15 °C over the course of trials (mean ± 1 sd, 12.5 ± 1.71 °C), in accordance with seasonal changes in lake temperature and discharge was maintained at or near 0.01 m^3^ s^−1^ in each raceway. Trials began by carefully releasing the fish into the center of the test area by opening the holding basket. Trials lasted 22 min, including a 5-min acclimation period, a 5-min pre-stimulus observation period, a 2-min period to initiate continuous pumping of the odor into the system and ensure it traveled through the test area, and then a 10-min stimulus observation period.

#### Experiment 2: Behavioral responses by groups in a large arena assay

Experimental arenas were isolated with block nets at upstream and downstream ends, forming a 3.1 m long reach (Fig. 1b). Arenas were lined with white plastic paneling (1/16in PLAS-TEX, Parkland Plastics, Inc., Middlebury, Indiana, USA) to increase visual contrast between sea lampreys and their background. A 1.2 m reach of HDPE paneling extended before the experimental arena (in the ‘odor mixing’ zone) to aid with stimulus partitioning. Arenas were illuminated with arrays of six infrared lights (Wildlife Engineering; Model IRLamp6) and experiments were recorded with overhead infrared sensitive video cameras (Axis Communications, Q1604 Network Camera). Water temperature ranged from 6 to 18 °C (mean ± 1 sd, 13 ± 1.73 °C) and discharge was maintained at 0.02–0.03 m^3^ s^−1^ in each experimental raceway. Trial groups consisted of 10 sea lampreys, held in the same holding baskets until the beginning of a trial. Each trial began by carefully releasing the ten animals from their holding basket into the middle of the experimental arena and lasted 30 min including a 10-min acclimation period and a 20-min observation period. Position data was collected during the acclimation period, where no odor was introduced. At the beginning of the observation period, the pumps were turned on and odor was introduced. No data was collected until the final 10-min of the observation period to allow the distribution of fish on the stimulus side to stabilize after odor introduction. This 10-min stabilization period was chosen based on a previous study that showed distribution of sea lamprey stabilized after 5-min of odor introduction^20^.

### Analyses

#### Experiment 1: Behavioral responses by individuals in a small-arena assay

Video analysis was completed in Behavioral Observation Research Interactive Software (BORIS), version 7.9.8^59^. In BORIS, all videos were watched in entirety and behaviors were manually recorded per the Ethogram in Table 1. Behaviors were scored based on the animal’s activity level, from low (1) to high (3) (Table 1). In low activity behaviors (score = 1), sea lampreys were not moving, with oral disks attached to the experimental arena (Table 1). In mid activity behaviors (score = 2), sea lampreys were actively moving and exploring the experimental arena at a nominal speed (Table 1). In high activity behaviors (score = 3), sea lampreys were moving at increased speeds and exhibiting behaviors such as darting, turning sharply in the open arena, and breaching the water with their heads. In BORIS, each time an animal switched from the odor to non-odor side (or vice versa) the change in position was recorded as an event. The ‘post exposure to stimulus’ period was only recorded after confirmation that the fish interacted with the cue after its addition into the assay, and thus a trial was discarded if a fish spent the entire observation period without moving into the stimulus odor. Two metrics of response were calculated: preference and change in activity. To analyze preference, the proportion of time a sea lamprey spent on the stimulus side was calculated by dividing the time spent in the stimulus by the total time after the first encounter with the stimulus. A proportion of time equal to 50% indicated neutral preference, and a distribution less than 50% indicated avoidance. All statistical analyses were done in R (Version 1.4.1103). A Shapiro–Wilk’s test showed that the data followed a non-normal distribution, and a Levene’s test confirmed unequal variances, thus a Kruskal–Wallis test was performed to test for any effect of odor treatment on avoidance response. A post-hoc Dunn’s test (α = 0.05) was completed as a means comparison of preference responses. To analyze activity level, each time a fish exhibited a behavior described in the Ethogram (Table 1) was recorded. An activity index was calculated using the activity score for each individual sea lamprey by multiplying the amount of time spent on each behavior by its activity level and combining as follows: (time spent on high activity behaviors * 3) + (time spent on medium activity behaviors * 2) – (time spent on low activity behaviors *1). Activity indexes were separately calculated for pre-exposure (during the pre-stimulus trial observation period) and post-exposure (during the stimulus trial observation period). To account for individual differences in baseline activity rate, the change in activity was calculated for each fish by subtracting the pre-exposure activity index from the post-exposure activity index (Δ activity). A significantly larger index score was evidence of increased activity after odor exposure. In R, a one-way ANOVA was performed with Δ activity index as the response variable and odor type as a fixed effect. Normality was confirmed with a Shapiro–Wilk’s test (α = 0.05) and Tukey’s Honestly Significant Difference (HSD) (α = 0.05) was completed as a post-hoc means comparison for each treatment to understand the effect of odor on activity.

**Table 1:** Ethogram used to analyze behavioral responses to odor treatments in BORIS software. Activity levels were used to calculate activity indexes.

Activity level	Description of behavior
1	Sea lamprey is unmoving and attached to the experimental arena with oral disk
2	Sea lamprey is active and exploring the arena at a nominal speed
3	Sea lamprey increases speed, frequent darting, sharp turns, and breaching of the surface are observed within the arena

#### Experiment 2: Behavioral responses by groups in a large arena assay

Videos of each trial were analyzed for preference responses by pausing every 30 s and counting the number of fish on each side of the raceway (stimulus or non-stimulus, Fig. 1b) as an indication of preference. Fish positions were designated based on the location of a fish’s head at each 30 s-time stamp. Positions were quantified during the pre-stimulus period (i.e., the 10-min acclimation period) and during the post-stimulus period, defined as the final 10 min of the observation period. This ensured that the introduced stimulus ran to the end of the experimental arena and allowed adequate time for the distribution of fish to stabilize after encountering an odor. Activity levels could not be scored in this experiment because individual fish within the group could not be identified from the video. All statistical analyses were done in R (Version 1.4.1103). A Shapiro–Wilk’s test confirmed data followed a non-normal distribution, and a Levene’s test confirmed unequal variances. A Kruskal–Wallis test was performed to test for any effect of odor treatment on avoidance response and post-hoc Dunn’s test (α = 0.05) was completed as a means comparison of preference responses. Putrescine, putrescine + petromyzonacil, and 10^–1^ M putrescine treatment means were compared to two alarm cue treatments (full body extract and crude skin extract) and solvent controls means to determine if putrescine elicited a partial, full, or not-significant avoidance response in sea lamprey.

## Results

### Experiment 1: Behavioral responses by individuals in a small-arena assay

#### Preference

**Figure 2:**
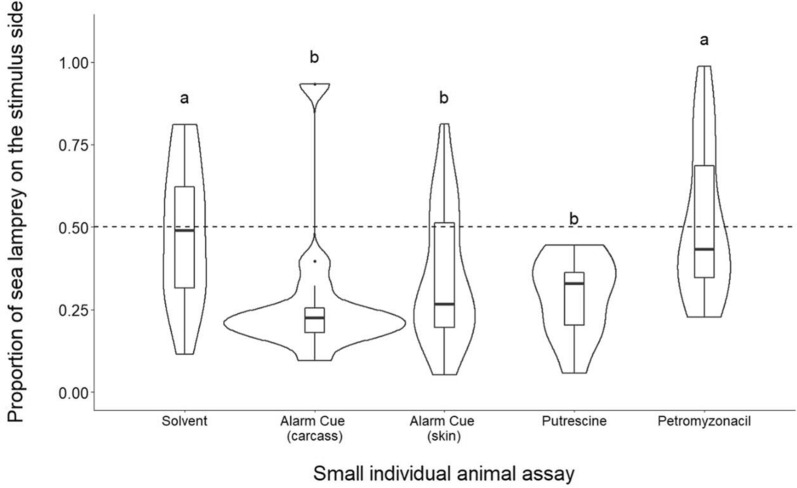
Boxplots representing the proportion of sea lamprey on the stimulus side after the addition of odorants. The middle quartile of boxes represents treatment median, and upper and lower quartiles are the 75th and 25th percentile of the range, respectively. Upper and lower whiskers represent the minimum and maximum spread of the data. Violin plots demonstrate the frequency of proportion values for each treatment. Dashed line at 0.50 represents the null hypothesis of a true neutral response to introduced stimulus. Treatments with different letters are significantly different from one another based on Dunn’s test (*α* = 0.05). N = 18 for solvent, N = 13 for full body alarm cue, N = 19 for skin alarm cue, N = 10 for putrescine and N = 19 for petromyzonacil.

Odor exhibited a significant effect on sea lamprey preference behavior (Kruskal-Wallace χ^2^ (4) = 17.78, *p* = 0.001). Each alarm cue treatment showed a significant avoidance response compared to the solvent control (Dunn’s test, full-body and skin *p* < 0.05; Fig. 2). Putrescine showed significantly higher avoidance response than the solvent (Dunn’s test, *p* = 0.04; Fig. 2), and was not significantly different than either alarm cue treatment (Dunn’s test, full-body *p* = 0.99 and skin *p* = 0.99; Fig. 2). Responses to petromyzonacil were not significantly different from the solvent (Dunn’s test, *p* = 0.74; Fig. 2), and sea lamprey spent more time in the odor than during the alarm cue treatments (Dunn’s test, full-body *p* < 0.001; skin *p* = 0.02).

#### Activity

**Figure 3:**
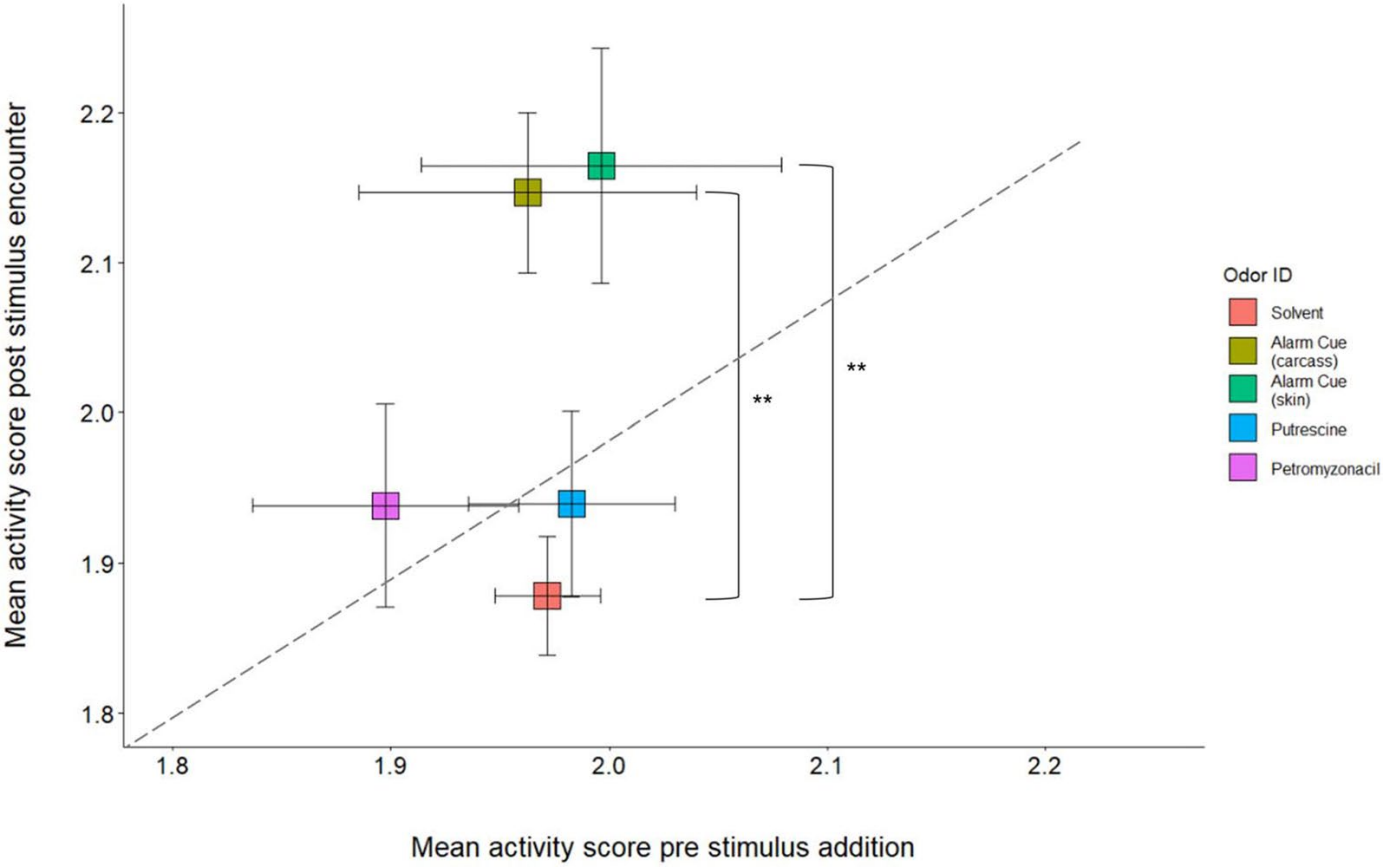
Mean (± 1SE) activity score before addition of odorants by mean (± 1SE) activity score after encounter with odorant. Responses to odors with an asterisk represent a significantly different change in activity (*p < 0.05; **p < 0.01; ***p < 0.001) based on Tukey HSD. The dashed line represents the null hypothesis of no change in activity before and after stimulus exposure. Responses above the line indicate an increase in activity post exposure to the odor, and responses below the line indicate a decrease in activity post exposure to the odor. The minimum score possible is 1 (indicating the entire trial period was spent on low activity behaviors per the ethogram in Table 1) and maximum is 3 (indicating the entire trial period was spent on high activity behavior per the ethogram in Table 1).

Odor exhibited a significant effect on the change in sea lamprey activity (ANOVA, *F*_4,74_ = 4.493, *p* < 0.01). Both alarm cue treatments showed a significant increase in activity compared to the solvent control (Tukey HSD, full-body *p* = 0.01; skin *p* < 0.01; Fig. 3). Petromyzonacil showed no significant difference compared to the solvent (Tukey HSD, *p* = 0.40; Fig. 3) or alarm cue treatments (Tukey HSD, full-body *p* = 0.43; skin *p* = 0.44; Fig. 3). Putrescine also showed no significant difference compared to the solvent (Tukey HSD, *p* = 0.98; Fig. 3) or alarm cue treatments (Tukey HSD, each *p* = 0.14; Fig. 3).

### Experiment 2: Behavioral responses by groups in a large arena assay

**Figure 4:**
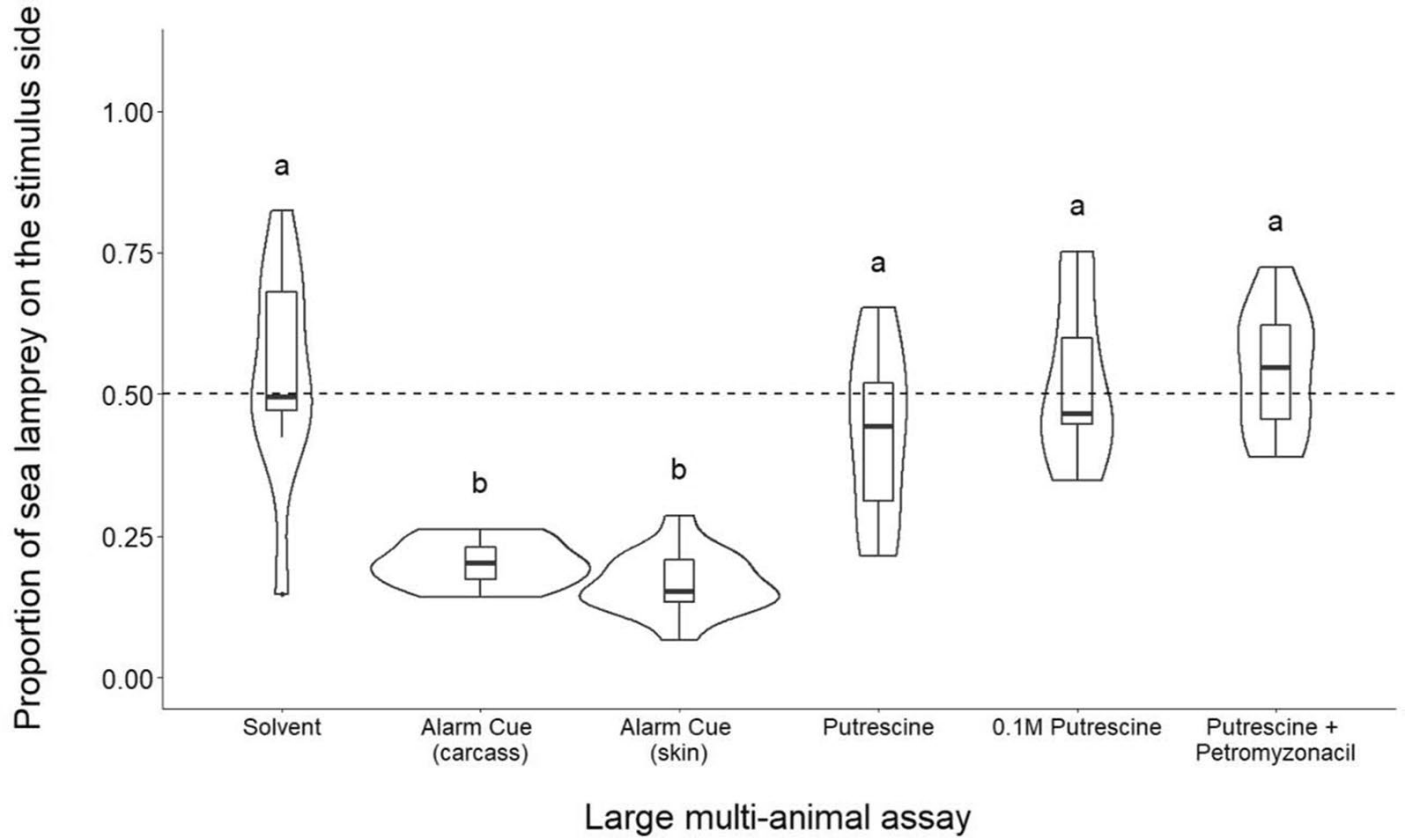
Boxplots representing the proportion of sea lamprey on the stimulus side after the addition of odorants. The middle quartile of boxes represents treatment median, and upper and lower quartiles are the 75th and 25th percentile of the range, respectively. Upper and lower whiskers represent the minimum and maximum spread of the data. Violin plots demonstrate the frequency of proportion values for each treatment. Dashed line at 0.50 represents the null hypothesis of a true neutral response to introduced stimulus. Treatments with different letters are significantly different from one another based on Dunn’s test (*α* = 0.05). N = 15 for crude skin alarm cue treatment. N = 10 for solvent, full body alarm cue, putrescine, and putrescine + petromyzonacil treatments. N = 5 for 10^–1^ M putrescine treatment.

The model results (Kruskal-Wallace χ^2^ (5) = 39.59, *p* < 0.001) clearly indicated that the type of odor introduced into the raceway channel influenced sea lamprey space use. The proportion of time spent on the stimulus side was significantly lower in both the full-body alarm cue treatment and the crude skin alarm cue treatment than the solvent treatment (Dunn’s test, full-body *p* < 0.01; skin *p* < 0.001; Fig. 4). Putrescine treatments failed to exhibit avoidance responses and were not significantly different from the solvent control (Dunn’s test, putrescine *p* = 0.99, putrescine + petromyzonacil *p* = 0.99, 10^–1^ M putrescine *p* = 0.99; Fig. 4).

## Discussion

Our results suggest migrating sea lamprey respond to putrescine, but the response is substantially less than that to alarm cue, only arises in certain contexts, and does not synergize with petromyzonacil, a unique compound found in sea lamprey skin. In small arenas (Experiment 1), individual sea lamprey strongly avoided conspecific alarm cues derived from full body or skin, and exhibited increased activity, often swimming near the surface and probing with its head out of the water in an apparent attempt to find egress from the arena (i.e., an overt escape response^60^). Similar responses have been reported in larval sea lamprey exposed to alarm cue in small tanks^61^. Individuals exposed to putrescine also exhibited avoidance, but exposure did not result in a significant increase in activity, suggesting an intermediate response more consistent the perception of lower risk, or an adaptive ‘disgust’ response affiliated with avoiding exposure to disease^62^. Given the weaker responses vs full body or skin alarm cue treatments, these results were more consistent with the hypothesis that putrescine operated as an indicator of decaying tissue that allows migrants to avoid scavenging predators and/or exposure to contagion. However, in a larger arena and in the presence of conspecifics (Experiment 2), exposure to putrescine failed to elicit an avoidance response. Taken together, these data support the contention that risk-sensitive decisions arise across odor classes that relate to differing types of threat, and the degree of confinement and/or social interaction may mediate responses to those threats.

An animal’s perception of risk is partly a result of the imminence of the threatening agent, a perception that can be modulated by the environment in which the information is received (e.g. predation threat per Fanselow and co-authors^52,63^) resulting in the expression of graded or threat-sensitive responses^64^. Here, we found that small arenas elicited an avoidance response to putrescine that was not evident in larger arenas. This strongly suggests that putrescine is less threatening than alarm cue and that the odorant elicits a chemosensory response as a source of decay- likely to avoid disease or scavenging predators. Certain fishes avoid infected conspecifics in a threat-sensitive manner^65^, and recent evidence suggest they can detect the odor of infectious microorganisms^66^. Trinidadian guppies (*Poecilia reticulata*) reduce shoal cohesion upon detection of infection^67^ and respond to visual cues of infected conspecifics at close range^65^, behaviors likely used to increase space between infected and non-infected individuals to reduce rates of disease transmission. This aligns with the observed avoidance pattern in sea lamprey in the small assays, where threat of disease would be more spatially imminent than in the larger assay.

Sea lamprey did not avoid any putrescine treatment in the large, multi-animal assay, which contrasts with well documented evidence of avoidance to alarm cue in raceway systems^10,19–22,25,43,68^, including evidence in the current study (Fig. 4). While the minimum detectable dosage of putrescine is unknown in sea lamprey, the activation concentration of putrescine in teleost fishes (zebrafish) is 4.0 µM^30^, which is well surpassed in the high concentration (10^–1^ M) treatment and the lower-concentration (10^–4^ M) putrescine treatments. While the sea lamprey olfactory sensory neurons (OSNs), are morphologically different than those of teleost fishes, they bear striking similarities, showing evidence of highly conserved OSN morphotypes in vertebrates^69^. In general, sea lamprey have proven substantially more sensitive to semiochemicals when compared to teleost fishes^70^. Thus, although putrescine was identified as a compound within the tissue extracts containing the alarm cue^39^, we generated no evidence to support the hypothesis that putrescine is part of the behaviorally active suite of compounds within this cue. Because the alarm cue mixture is extracted from dead organisms, the nature of putrescine within the mixture is unknown and may be present as a part of natural decomposition.

There also was limited support for the hypothesis that putrescine was the principal salivary kairomone. In the larger, multi-animal assay, we did not find evidence that putrescine avoidance responses were similar to human saliva in studies that used similar assay designs^10,42,43^. Human saliva is a mixture, and it is possible that putrescine is part of this mixture. However, our data shows little evidence that putrescine elicits the expected spit response, such as avoidance or increased activity, on its own. In 1809, Tilden^41^ described lamprey jumping out of water in “great agitation” when human saliva was introduced, a pattern which was not seen in activity change within the small individual assay (Fig. 3), and previous studies found a consistent and significant avoidance of human saliva in larger multi-animal assays^10,42,43^. We did record a significant avoidance pattern in the small assay, of different design than prior studies (Fig. 2), suggesting the avoidance response to putrescine is context specific in sea lamprey. Future studies should investigate putrescine in combination with other known components of human saliva already identified^71^. The response to other mammalian saliva samples, especially of those known to prey on sea lamprey, such as raccoons and river otters^10^, should also be investigated to understand the role of mammalian saliva as a putative predator kairomone, and any overlaps in chemical composition should be identified. Tilden’s 1809 account^41^ reported that lamprey did not elicit any behavioral response to dog saliva, and the possibility remains that human saliva contains similar reactive compounds to the alarm cue and elicits an anti-predator response by happenstance.

The smaller arena may also have affected the animal’s perception of safety compared to the larger multi-animal assay. It has been shown that aquatic organisms respond both to perceptions of fear (threat level) and safety (vulnerability to a threat) when making movement decisions (e.g., crayfish, *Faxonius rusticus*^72^). It is plausible that the larger multi-animal assay provided more cues of safety than the small individual assay. Both assays were relatively shallow, and sea lamprey may perceive a shallow environment as riskier because of increased vulnerability to shoreline predators^10^. Sea lampreys are hypothesized to use water depth via hydrostatic pressure detection to orient towards shallower waters during the start of their spawning migration^73^.There also is some evidence that they may prefer to migrate in the deeper thalweg of shallow streams^74^. Despite the shallow conditions, the larger arena had an area three times that of the smaller arena (Fig. 1a,b), which likely provided animals with an increased perception of safety, because at any one point in the arena there is a larger area/distance available to move away from a threat compared to the smaller assay. A fish’s social context can also affect risk perception. Trinidadian guppies from high predation environments move in more cohesive shoals than those from low predation environments^75^, and after exposure to alarm cues x-ray tetras (*Pristella maxillaris*) increase observation and responsiveness to group members^76^. Similarly, banded killifish (*Fundulus diaphanous*) form larger group sizes when exposed to alarm cues than when exposed to food cues^77^. However, there is no evidence that sea lamprey socially aggregate or shoal during their upstream spawning migration, as multi-year assessments of springtime stream entry into Lake Huron indicate solitary movement patterns^46^. Siefkes et al.^78^ also found no evidence of following behavior when observing sexually mature female sea lamprey tracking a pheromone plume to its source. These findings are expected, as migratory sea lamprey are nocturnal and have no obvious mechanism to maintain contact with conspecifics in complete darkness. We also observed no evidence of group behavior nor individuals reacting to each other (e.g. synchronized turning) in the present study. However, many aquatic organisms are known to produce and respond to disturbance cues, which are distinct from alarm cues in that they are volitionally emitted upon the perception of risk and act as social cues^14,16,79^. Traditional ecological knowledge from Karuk and Yurok tribe fishers suggests the Pacific lamprey (*Entosphenus tridentatus*) emits a disturbance cue when handled, initiating a downstream flight response in other migrants^80^. Thus, while it is unlikely that the sea lamprey uses direct observations of conspecifics when selecting anti-predator tactics, it is possible that the inclusion of more animals in Experiment 2 may have provided an indirect measure of safety (i.e., the absence of disturbance cues), as individuals could gain information of risk from other individuals in the arena. If sea lamprey do produce a disturbance due in response to perceived risk, this would lend further credence to the hypothesis that putrescine is not a risk-related cue for sea lamprey, as such releases should have induced alarm responses in the group trials.

Confinement can also lead to stress which may have affected the observed differences between assays^81^. Metabolic changes associated with stress can be significant in mediating anti-predator behaviors^82,83^, but there have been few reported studies testing this hypothesis in fishes^82^. Anti-predator responses have been linked to increased cortisol levels in Nile tilapia (*Oreochromis niloticus*)^84^, coho salmon (*Oncorhynchus kisutch*)^85^, and freshwater pearl dace (*Margariscus margarita*)^86^. A similar corticosteroid, 11-deoxycortisol, has recently been shown to play a role in sea lamprey gluconeogenesis, evidence that corticosteroid function is present in basal vertebrates^87,88^. However, both checkered puffer fish (*Sphoeroides testudineus*)^89,90^ and schoolmaster snapper (*Lutjanus apodus*)^82,91^ did not show evidence of increased anti-predator behaviors with increased cortisol levels. The influence of stress is likely context specific, and more research is needed on a variety of contexts to understand complex interactions between internal stress states and anti-predator behaviors^82,84^. It has been recommended that the size of a two-choice arena in studies with fishes should be decided based on the size of the species using the assay. Assays for larger, more motile fish should be designed to allow sufficient areas for movement and exploration and to minimize confinement stress^92–94^. The animals used in the study are notably motile during this life stage, as they actively migrate over large distances in search of suitable spawning habitat^95^; thus, a larger arena may prove less stressful for sea lamprey. One review suggested that the width and length of an experimental arena should be approximately 4–15 times the length of the organism, based on over a dozen studies of aquatic animals in two-choice assay experiments^94^. The average length of sea lamprey used in this study was 0.47 m, and the area of the experimental arena used in the small arena assay was 1.22 m × 1.22 m (i.e., 2.6 times the average length of experimental subjects, below the recommended threshold).

We also investigated the reaction to petromyzonacil, a novel molecule identified from sea lamprey skin^55^ on its own and in combination with putrescine. This compound was investigated as a potential species-specific labeling compound, as it has been hypothesized that the alarm cue is a mixture containing compounds that indicate risk and others that label the species giving rise to the risk cue, consistent with evidence that responses to heterospecific alarm cues diminish with increased phylogenetic distance^19^. Here, we saw no effect on sea lamprey preference or activity in treatments with petromyzonacil on its own (Fig. 2) and found no synergistic effects with putrescine in the large assay (Fig. 4). Therefore, there is no evidence based on behavioral screening that this molecule is behaviorally reactive at the concentrations tested.

In the small assay, we observed significant avoidance and increases in activity in response to both alarm cue treatments as previously reported in laboratory^10,18,19,21,43^ and field studies^20,25,68,96^. Thus, this individual assay may be a useful tool to investigate activity and behavioral patterns to odor stimuli. One particular benefit to this assay is its high-throughput design, achieved in two ways: (1) the assay build is smaller, so more apparatuses can be built side-by-side into raceways or natural streams and run simultaneously; and (2) trial time is cut down, so more trials can be completed in one night. A high-throughput, individual design requires fewer animals to achieve statistical significance, as each animal represents a replicate. It also requires less odor material, as there are more replicates per unit of odor. The individual assay can also allow researchers to investigate complex questions in inter-individual variation of behavioral patterns related to animal personality, an area of research which has been identified as particularly important to bridge basic and applied research in conservation studies^97^. To improve assay design and understand its efficacy in behavioral research, we suggest future research to investigate how sea lamprey activity is affected by varying arena dimensions to understand how size effects the animal’s perception of vulnerability. We also suggest studies investigating behavioral risk avoidance patterns in sea lamprey in assays with differing depths to understand how water depth affects the organism’s perception of vulnerability. While a smaller assay does not represent how a sea lamprey would respond to stimuli in nature, it could be used as a fruitful screening tool to then be scaled up to larger raceway or field studies.

In sum, we found limited evidence of repellent activity to putrescine in migratory sea lamprey after two experiments investigating avoidance and activity change to the odorant. Our work showed the potential efficacy of a small scale, individual animal behavioral assay as a screening tool for behavioral and chemical ecology research. Within the context of the predatory imminence hypothesis, our results showed support that putrescine is most likely perceived as a death, or ‘rot’, odor in the sea lamprey lifecycle as the threat of disease may be more imminent in smaller arenas. We found limited support that putrescine acts a predator odor, or kairomone, because the avoidance response in the large arena was not consistent with human saliva studies in similar assays^10,43^, and found lowest support that putrescine acts as the active compound of the alarm cue because it did not change activity or elicit an avoidance response in the larger assay. Future research should continue to investigate how assay metrics constrain behavior and continue to research potential repellent molecules, including other decay molecules such as cadaverine, for use in management of invasive aquatic species such as the sea lamprey. More research needs to be done to understand the response of sea lamprey to saliva, with the most notable gap in the avoidance response to saliva of mammalian predators. The overlap of reactive compounds between alarm cue and human saliva should be identified to understand if saliva acts as a predator kairomone for migratory sea lamprey, or simply overlaps in chemical character with the alarm cue (Supplementary Information S1).

## Supplementary Information

Supplementary Information 1.

Supplementary Information 2.

Supplementary Information 3.

## Data Availability

Data is available as Supplemental Dataset files or by request to E.L.M or C.M.W.
